# Sonication contribution to identifying prosthetic joint infection with *Ralstonia pickettii*: a case report and review of the literature

**DOI:** 10.1186/s12891-017-1678-y

**Published:** 2017-07-19

**Authors:** Rares Mircea Birlutiu, Mihai Dan Roman, Razvan Silviu Cismasiu, Sorin Radu Fleaca, Crina Maria Popa, Manuela Mihalache, Victoria Birlutiu

**Affiliations:** 1 Lucian Blaga University of Sibiu, Faculty of Medicine Sibiu; FOISOR Clinical Hospital of Orthopedics, Traumatology, and Osteoarticular TB Bucharest, Address: Str. Lucian Blaga, Nr. 2A, 550169 Sibiu, Romania; 2 Lucian Blaga University of Sibiu, Faculty of Medicine Sibiu, Academic Emergency Hospital Sibiu - Orthopedics and Traumatology Department, Address: Str. Lucian Blaga, Nr. 2A, 550169 Sibiu, Romania; 3 Carol Davila University of Medicine and Pharmacy Bucharest, Romania; FOISOR Clinical Hospital of Orthopedics, Traumatology, and Osteoarticular TB Bucharest, Address: Str. Dionisie Lupu nr. 37, 020021 Bucharest, Sector 2 Romania; 4 Polisano European Hospital Sibiu, Address: Str. Izvorului Nr. 1A, Sibiu, Romania; 5 Lucian Blaga University of Sibiu, Faculty of Medicine Sibiu, Address: Str. Lucian Blaga, Nr. 2A, 550169 Sibiu, Romania; 6 Lucian Blaga University of Sibiu, Faculty of Medicine Sibiu, Academic Emergency Hospital Sibiu - Chief of the Infectious Diseases Departmen, Address: Str. Lucian Blaga, Nr. 2A, 550169 Sibiu, Romania

**Keywords:** *Ralstonia pickettii*, Prosthetic joint infection, Sonication, Biofilm, Spacer, Case report

## Abstract

**Background:**

In the context of an increase number of primary and revision total hip and total knee arthroplasty performed yearly, an increased risk of complication is expected. Prosthetic joint infection (PJI) remains the most common and feared arthroplasty complication. *Ralstonia pickettii* is a Gram-negative bacterium, that has also been identified in biofilms. It remains an extremely rare cause of PJI. There is no report of an identification of *R. pickettii* on an extracted spacer loaded with antibiotic.

**Case presentation:**

We present the case of an 83-years-old Caucasian male patient, that underwent a right cemented total hip replacement surgery. The patient is diagnosed with an early PJI with no isolated microorganism. A debridement and change of mobile parts is performed. At the beginning of 2016, the patient in readmitted into the Orthopedic Department for sever, right abdominal and groin pain and elevated serum erythrocyte sedimentation rate and C-reactive protein. A joint aspiration is performed with a negative microbiological examination. A two-stage exchange with long interval management is adopted, and a preformed spacer loaded with gentamicin was implanted. In July 2016, based on the proinflammatory markers evolution, a shift a three-stage exchange strategy is decided. In September 2016, a debridement, and changing of the preformed spacer loaded with gentamicin with another was carried out. Bacteriological examination of the tissues sampled intraoperatively was positive for *Pseudomonas aeruginosa*. From the sonication fluid, no bacteria were isolated on culture or identified using the bbFISH assay. During the hospitalization period, the patient received i.v. ceftazidime 3x2g/day and p.o. ciprofloxacin 2x750mg/day, antibiotic therapy that was continued after discharge with p.o. ciprofloxacin 2x750mg/day for 6 weeks. In February 2017, a reimplantation of a revision prosthesis is performed. The retrieved spacer is sonicated, and after 4 days of incubation of the sonication fluid, *R. pickettii* is isolated. A long term antibiotic therapy with cotrimoxazole being prescribed.

**Conclusions:**

Bacteria culture of sonication fluid remains the gold standard in diagnosing prosthetic joint infections. *R. pickettii* remains an extremely rare cause of prosthetic joint infection. Optimal management of *R. pickettii* prosthetic joint infections of has not been established.

## Background

Orthopedic surgery of total hip and total knee arthroplasty were the most successful orthopedic surgeries of the last century. In the context of an increase number of primary and revision total hip and total knee arthroplasty performed each year, there it is expected an increased risk of complication. Prosthetic joint infection (PJI) remains the most common and feared arthroplasty complication. A correct diagnosis of infection is decisive for a correct treatment of orthopedic implant-related infections.

The diagnosis of PJI is based on history of the patient (fever, pain, surgeries in the past), clinical assessment (the presence of a sinus tract) and laboratory tests. The laboratory criteria of PJI diagnosis have not yet been established.

*Ralstonia pickettii*, is a Gram-negative bacilli, rod-shaped, non-fermentive, soli bacterium, that can cause severe nosocomial infections, in immunocompromised patients (premature infants, Crohn’s disease, cystic fibrosis, leukemia), like meningitis, sepsis, pneumonia, and osteoarticular infection through contamination of the sterile water, saline solution, disinfectants, etc. Prosthetic joint infection caused by *R. pickettii* is rarely reported in literature, a few cases have been described, there is no report of an identification of *R. pickettii* on an extracted spacer loaded with antibiotic (gentamicin).

## Case presentation

We present the case of an 83-years-old Caucasian male patient, known with high blood pressure, mitral valve regurgitation, coronary artery disease, diabetes mellitus type 2, that underwent a right cemented total hip replacement surgery for osteoarthritis in 2014 in the Academic Emergency Hospital Sibiu, Romania. Detailed clinical patient information is given in Table 1. Three weeks after the surgery, the patient is diagnosed with an early PJI with no isolated microorganism from periprosthetic tissues or fluids harvested during the surgery. A debridement and change of mobile parts with the retention of fixed prosthetic components is performed. Soft tissues surrounding the implant and periprosthetic interface membrane were taken for histopathological testing. The patient was discharged from the hospital and treated as an outpatient with cefuroxime 2 × 500 mg/day for 2 weeks.

**Table 1:** Clinical details of the patient

Date and type of surgery	November 2014 Debridement, and retention of fixed prosthetic components	March 2016 Debridement, explanation of the prosthesis, and implantation of preformed spacer loaded with gentamicin^a^	June 2016 Internal Medicine Department Hospitalization	July 2016 Debridement, and changing of the preformed spacer loaded with gentamicin	September 2016 Debridement, and changing of the preformed spacer loaded with gentamicin	February 2017 Debridement, and reimplantation of a revision prosthesis
Blood sugar mg/dl (R.V. 83–110)	190	139	139	109	239	142
Fibrinogen mg/dl (R.V. 180–400)	530.5	424.7	438.2	523.2	560	388.9
WBCs (R.V. 4–10 × 103/μl)	8.46	8.9	8.33	11.07	9.11	4.96
HGB (R.V.13-17 g/dl)	10.5	11.6	11.5	11.0	9.4	11.7
NEUT (R.V. 2–7.5 × 103/μl)	6.16	6.1	6.47	8.38	7.61	3.31
ESR (R.V. 0-15 mm/h)	69	27	22	47	46	16
C-reactive protein (R.V. <6 mg/dl)	NA	27	22	24	24	6
Joint fluid hs-CRP (R.V. 0.00–0.50 mg/dl)	NA	NA	-	NA	0.78	0.89
Histopathology Results^b^	Type II	Type III	-	Type II	Type II	Type II
Culture results						
Preoperative samples (joint fluid)	Negative	Negative	-	Negative	Negative	Negative
Intraoperative samples (tissue biopsies – 3 samples)	Negative	*Pseudomonas aeruginosa* (in all samples)	-	Negative	*Pseudomonas aeruginosa* (in 2 samples)	*Pseudomonas aeruginosa* (in 1 sample)
Sonication fluid culture	NA	NA	-	NA	Negative	*Ralstonia pickettii*
Molecular identification of bacteria by 16S rRNA bbFISH® technology^c^						
On Sonication Fluid	NA	NA	-	NA	Negative	Negative

^a^Signs of femoral and acetabular component loosening were present; ^b^ according to the Krenn and Morawietz [2]: type I periprosthetic membrane (ppm) of the wear particle-induced type, type II ppm of the infectious type, type III ppm of the combined type, type IV indeterminate type; ^c^ Beacon-based fluorescent in situ hybridization kit (hemoFISH® Masterpanel, miacom diagnostics GmbH Düsseldorf, Germany); *R.V* references values, *WBC* White blood cells, *HGB* hemoglobin, *NEUT* neutrophils, *ESR* erythrocyte sedimentation rate, *NA* not available

At the beginning of 2016, the patient is readmitted into the Orthopedic Department for sever, right abdominal and groin pain, and elevated serum erythrocyte sedimentation rate and C-reactive protein. An anteriorposterior (AP) radiograph of the pelvis and a horizontal beam lateral hip radiograph of the right hip are performed, reveling signs of loosening at the level of both the acetabular and femoral components. A joint aspiration is performed with a negative microbiological examination. A two-stage exchange with long interval management is adopted, and a preformed spacer loaded with gentamicin was implanted. Intraoperative tissue samples are harvested and *Pseudomonas aeruginosa* is isolated. Oral ciprofloxacin 2 × 750 mg/day is administered for 6 weeks and intravenous (i.v.) gentamicin 1 × 240 mg/day for 2 weeks after the discharge from the hospital, according to the results of the susceptibility tests that were performed.

The patient was readmitted in one of the Internal Medicine Department, after 3 months, for right groin pain and altered proinflammatory markers, for which nonsteroidal anti-inflammatory drugs, muscle relaxants, and anti-clotting drugs are administered with favorable outcome.

In July 2016, based on the proinflammatory markers evolution, a shift a three-stage exchange strategy is decided. A joint aspiration is performed with a negative microbiological examination. A debridement, and changing of the preformed spacer loaded with gentamicin with another was carried out. Intraoperatively soft tissues were taken for histopathological testing. Oral ciprofloxacin 2 × 500 mg/day is administered for 3 weeks, according to the results of the susceptibility tests that were performed.

With the beginning of September 2016, a new strategy for the diagnostics and management of prosthetic joint infection is implemented in the Academic Emergency Hospital Sibiu. Strategy that includes the sonication of the retrieved implant (prosthesis or polymethylmethacrylate spacer). In the operating theater, Ringer’s or saline sterile solution in added in the sterile containers. The implants are processed within 30 min by sonication (1 min) using an ultrasound bath (BactoSonic®14.2, Bandelin GmbH, Berlin, Germany) at a frequency of 42 kHz and a power density of 0.22 W/cm^2^. The resulting sonication fluid is vortexed, and 50 ml of sonication fluid is centrifuged at 2500 rpm for 5 min. The resulted precipitate is inoculated onto Columbia agar with sheep blood (incubated aerobically, anaerobically and in high concentration of CO_2_), Sabouraud plate, MacConkey agar plate, glucose broth, lactose broth and thioglycollate broth. Cultures are incubated at 37 °C for 14 days and inspected daily for bacterial growth. Isolated bacteria are identified using the VITEK 2 Compact analyzer (bioMérieux, Marcy-l’Étoile, France). The MICs (minimum inhibitory concentrations) are assessed according to the European Committee on Antimicrobial Susceptibility Testing breakpoints. In addition, as a rapid method of bacteria detection, molecular identification of bacteria by 16S rRNA bbFISH (beacon-based fluorescent in situ hybridization) technology using a bbFISH kit (hemoFISH® Masterpanel, miacom diagnostics GmbH Düsseldorf, Germany), was also implemented. The FISH assay are performed according to the manufacturer using a sample of resulted precipitate from 50 ml of sonication fluid that is centrifuged at 2500 rpm for 5 min. The kit contains beacons for the detection of *Staphylococcus spp.*, *Staphylococcus aureus*, *Streptococcus spp.*, *Streptococcus pneumoniae*, *Streptococcus agalactiae*, *Enterococcus faecium*, *Enterococcus faecalis*, *Enterobacteriaceae*, *Escherichia coli*, *Klebsiella pneumoniae*, *Proteus mirabilis*, *Pseudomonas aeruginosa*, *Acinetobacter spp.*, and *Stenotrophomonas maltophilia.*

In September 2016, a joint aspiration is performed with a negative microbiological examination. A debridement, and changing of the preformed spacer loaded with gentamicin with another was carried out. Soft tissues surrounding the implant and periprosthetic interface membrane were taken intraoperatively for histopathological testing. Bacteriological examination of the tissues sampled intraoperatively was positive for *Pseudomonas aeruginosa*, strain that was identified using the VITEK 2 Compact analyzer (bioMérieux, Marcy-l’Étoile, France) and was sensitive to amikacin, cefepime, ceftazidime, ciprofloxacin, pefloxacin, Piperacillin, ticarcillin, meropenem, imipenem, colistin, gentamicin resistant to trimethoprim/sulfamethoxazole. From the sonication fluid, no bacteria were isolated on culture or identified using the bbFISH assay. During the hospitalization period, the patient received i.v. ceftazidim 3 × 2 g/day and p.o. ciprofloxacin 2 × 750 mg/day, antibiotic therapy that was continued at home after discharge with p.o. ciprofloxacin 2 × 750 mg/day for 6 weeks.

After 5 months, in February 2017, based on the serum proinflammatory markers levels and after a joint aspiration was performed with a negative microbiological examination, a debridement, and reimplantation of a revision prosthesis is performed. Soft tissues surrounding the implant and periprosthetic interface membrane were taken intraoperatively for histopathological testing. Bacteriological examination of the tissues sampled intraoperatively was positive for *Pseudomonas aeruginosa*, strain with the same susceptibility as the one isolated earlier. The retrieved spacer is sonicated. From the sonication fluid, after 4 days form the inoculation of the sonication fluid on the growth medium, *Ralstonia pickettii* was isolated. The isolated strain of *R. pickettii* was sensitive to ticarcillin, piperacillin, cefepime, imipenem, meropenem, ciprofloxacin, pefloxacin, minocycline, cotrimoxazole, resistant to aztreonam, amikacin, gentamicin, colistin, and intermediate sensitive to ceftazidime. No bacteria were identified using the bbFISH assay. The antibiotic therapy with p.o. cotrimoxazole 3 x 2tb (80 mg trimethoprim bp and 400 mg sulfamethoxazole bp)/day is initiated during the hospitalization period, and will be administered for a total duration of 12 weeks, the patient is discharged after 10 days from the admission into the hospital and is scheduled for routine follow-ups. On discharge, the wound is healed, with no open areas, and with sutures that are intact (and will be removed at 21 days after the surgery). The wound edges are well-approximated. The skin surrounding the wound is viable and clean. The wound looks good with no drainage. The dressing was clean and dry. The patient evolution, undertreatment, is favorable. At the last follow-up visit at (14 weeks after the surgery) the wound is healed, with no open areas, and with a clean skin surrounding the wound. The patient is using a cane for long walks, and with no clinical complaints. The laboratory investigations (white blood cells count, fibrinogen, C-reactive protein, erythrocyte sedimentation rate, creatinine/estimated glomerular filtration rate (eGFR), liver enzymes (aspartate aminotransferase - AST/SGOT and alanine aminotransferase - ALT/SGPT)) were performed on regular base and were within references levels.

## Discussions

*Ralstonia pickettii*, also known previously under other names like *Burkholderia pickettii*, *B. solanacearum*, *Alcaligenes eutrophupickettii* is part of Betaproteobacteria class, Burkholderiales order, Ralstoiaceae family, Ralstonia genus, is a Gram-negative, nonfermentative, oxidase-positive bacteria. The genus Ralstonia includes 10 other bacteria: *R. insidiosa* [1], *R. solanacearum*, *R. mannitolilytica* [2], *R. eutropha*, *R. gilardii*, *R. paucula*, *R. basilensis*, *R. oxalate*, *R. taiwanensis*, *R. campinensis*, and *R. metallidurans* [3–7]. It is an opportunistic pathogen associated with nosocomial infections due to contamination of sterile water, saline solution [8, 9], disinfectants, blood culture bottles, and venous catheters. Exceptionally being isolated from the mouth and upper respiratory tract [4], being responsible for lung abscess after necrotizing pneumonia in elderly, non-hospitalized patients [10] or lobar pneumonia associated with severe respiratory insufficiency [11].

*R.pickettii* is present in the environment, in water, plants, in enriched in heavy metals soil; may be associated in biofilm, being able to grow in nutrient deficient conditions. It can affect immunocompromised hosts, especially patients with Crohn’s disease or cystic fibrosis [12]. So far, around 70 cases of infection caused by *R. pickettii*, in humans, have been published in the literature. There are described cases of systemic infections in premature infants like bacteremia [13], infection associated with the administration contaminated heparin of flush [14], infection that tend to evolve, potentially severe, in infants, with severe sepsis, multiple organ failure, and disseminated intravascular coagulation [15]. *R. pickettii* bacteremia, was associated also with the use of contaminated antiseptic solutions - chlorhexidine solutions, and distilled water or with extracorporeal membrane oxygenation [16], and with the contamination of irrigation system in obstetric care [17]. *R. pickettii* was identified also in septic arthritis, spinal osteitis [18], osteomyelitis [19], meningitis [20] or ventricularperitoneal shunt infection [21]. In adults, fatal cases have been described in hematological patients (e.g. patients with leukemia) with immunosuppressive therapy and in patients with thalassemia that develop infection [22]. *R. pickettii* infection was also identified in patients with cochlear implants [23]. Identification in the male genital area appears to be associated with secondary infertility [24]. According to the data published in the literature, orthopedic implant-associated infection rate is between 1 and 4% for primary total joint arthroplasty [25, 26], nearly doubling (7%) for revision surgeries [25]. There are few cases of PJIs in which *R. pickettii* was isolated: Gomez-Barrena E et al. [27] identified 3 cases of infections associated with total knee arthroplasty, Lepetsos et al. published a case knee PJI [28], and Przemysław L [29], confirms the involvement in 3 cases of PJI, two knees and one hip PJIs. Esteban J et al. [30] identifies infections caused by *R. pickettii* after using sonication on intramedullary nails. A similar case like ours, in which *R. pickettii* to associated with an antibiotic spacer, was not published so far in the literature. According to other studies [31], identification of *R. pickettii* after sonication of the implant was superior to joint fluid cultures. In our case, the lack of identifying the strain in the previous step of the revision surgery, in which sonication was also used, suggests a potential recent infection, possibly being associated with the saline lavage during the debridement stage of the surgery. The antibiotic sensitivity, of the isolated strain, was at quinolones and trimethoprim/sulfamethoxazole, antibiotics used to treat the patient. The resistance of the strains to gentamicin, that was found in the spacers loaded with antibiotics, explains the growth of *R. pickettii* at this level. It is necessary to better assess in terms of bacteriological examination of the prosthesis, by sonication and molecular detection, and in terms of the local treatment options, options that should be efficient also against opportunistic pathogens resistant to aminoglycosides, or vancomycin, antibiotics that are commonly used in revision surgeries.

## Conclusions

In conclusion, bacteria culture of sonication fluid remains the gold standard in diagnosing prosthetic joint infections. Negative culture of preoperative joint aspiration and soft tissues surrounding the implant and periprosthetic interface membrane obtained intraoperatively, do not exclude the presence of bacteria on the implants. *Ralstonia pickettii* remains an extremely rare cause of prosthetic joint infection. The introduction of ciprofloxacin in spacers in association with aminoglycosides or vancomycin, seems to be a better option to extend the antibacterial effectiveness against opportunistic germs.

## Abbreviations

ALT/SGPT: alanine aminotransferase
AP: Anteriorposterior
AST/SGOT: aspartate aminotransferase
bbFISH: Beacon-based fluorescent in situ hybridization
eGFR: creatinine/estimated glomerular filtration rate
i.v: Intravenous
MICs: Minimum inhibitory concentrations
PJI: Prosthetic joint infection
